# Digital Dashboard Design Using Multiple Data Streams for Disease Surveillance With Influenza Surveillance as an Example

**DOI:** 10.2196/jmir.1658

**Published:** 2011-10-14

**Authors:** Calvin KY Cheng, Dennis KM Ip, Benjamin J Cowling, Lai Ming Ho, Gabriel M Leung, Eric HY Lau

**Affiliations:** ^1^School of Public HealthThe University of Hong KongHong KongChina

**Keywords:** Dashboard, dissemination, surveillance, influenza

## Abstract

**Background:**

Great strides have been made exploring and exploiting new and different sources of disease surveillance data and developing robust statistical methods for analyzing the collected data. However, there has been less research in the area of dissemination. Proper dissemination of surveillance data can facilitate the end user's taking of appropriate actions, thus maximizing the utility of effort taken from upstream of the surveillance-to-action loop.

**Objective:**

The aims of the study were to develop a generic framework for a digital dashboard incorporating features of efficient dashboard design and to demonstrate this framework by specific application to influenza surveillance in Hong Kong.

**Methods:**

Based on the merits of the national websites and principles of efficient dashboard design, we designed an automated influenza surveillance digital dashboard as a demonstration of efficient dissemination of surveillance data. We developed the system to synthesize and display multiple sources of influenza surveillance data streams in the dashboard. Different algorithms can be implemented in the dashboard for incorporating all surveillance data streams to describe the overall influenza activity.

**Results:**

We designed and implemented an influenza surveillance dashboard that utilized self-explanatory figures to display multiple surveillance data streams in panels. Indicators for individual data streams as well as for overall influenza activity were summarized in the main page, which can be read at a glance. Data retrieval function was also incorporated to allow data sharing in standard format.

**Conclusions:**

The influenza surveillance dashboard serves as a template to illustrate the efficient synthesization and dissemination of multiple-source surveillance data, which may also be applied to other diseases. Surveillance data from multiple sources can be disseminated efficiently using a dashboard design that facilitates the translation of surveillance information to public health actions.

## Introduction

Respiratory viruses cause significant global mortality and morbidity each year. Influenza virus is of particular public health concern due to its association with severe infections and deaths [1,2]. Disease surveillance provides useful information that helps monitoring trends and disease burden, planning, implementing, and evaluating appropriate prevention and control interventions as well as allocating resources [3]. At present, the World Health Organization (WHO) runs the largest human influenza virus surveillance network [4], which includes 136 institutions from 106 countries mainly focusing on the genetic and antigenic characteristics of prevailing strains. While the original purpose of WHO’s global influenza surveillance network was to recommend the content of the influenza vaccine for the subsequent influenza season [5], local and national prospective influenza and influenza-like illness surveillance systems also provide important information to policy makers and public health practitioners for situational awareness during periods of influenza activity.

Great strides have been made exploring and exploiting new and different sources of respiratory disease data, especially after the severe acute respiratory syndrome (SARS) epidemic in 2003 [6,7] and the 2009 influenza A (H1N1) pandemic. However, as different surveillance methods target only specific target groups of population possibly at different stages of disease progression, thus providing related but different information [8], using a single surveillance data stream to reflect overall disease activity may not be appropriate. It remains challenging to incorporate multiple surveillance data and fully utilize the available information from the data to summarize the overall situation.

On the other hand, there has been rather less research in the area of data dissemination, while the feedback of contextualized data to stakeholders is and must be a key aspect of any surveillance system if confidence with and enthusiasm for the system is to be maintained [9,10]. It is also important that the dissemination is not neglected. No matter how accurate and timely the upstream surveillance data collection and analysis of the surveillance system are, the return for the time and effort invested would be discounted if the means of health communication for data dissemination were suboptimal. Poor communications could lead to delayed information transfer, loss of information, or even misinterpretation of the surveillance results. Effort should also be taken for data dissemination to complete the surveillance-to-action loop [3]. 

Digital dashboards (also known as executive dashboards) describe computerized interactive tools typically used by managers to visually ascertain the status (or "health") of their business via key performance indicators. These tools emerged from the concepts of decision support systems in the 1970s, and, with the rapid growth in information technology through the 1990s, these have developed into standard tools in executives offices [11,12]. Their use has expanded to the field of medical science, for example, displaying bed occupancy, the availability of clinical staffs for hospital management [13,14], or monitoring the stock of different pharmaceutical items in the dispensary [15]. In a more general sense, digital dashboards simply describe systems for the visual presentation of key indicators in appropriate context, allowing rapid interpretation by an end user. A dynamic dashboard can be made interactive and user-friendly by allowing extraction of information in different perspectives and contexts. By investing in the design of data dissemination, the quality of presentation and communication of data by local health authorities as well as the general public could be much improved, thus maximizing the usefulness of available data of the surveillance systems.

In this research paper, we developed a generic framework for a digital dashboard and illustrate this framework by specific application to influenza surveillance. Specific features were designed and incorporated according to the requirements identified to facilitate efficient dissemination of surveillance data. The dashboard can reveal both the influenza activity in different population sectors and the overall situation at a glance, maximizing the use of available data for policy makers as well as the general public for appropriate actions.

## Methods

### Rationale and Implementation of the Dashboard Design

We designed an online interactive dashboard that fit seamlessly with human visual perception. Three authors of the current study (CKYC, BJC, EHYL), with backgrounds in computer science, epidemiology, and statistics, identified weaknesses of current surveillance data dissemination methods according to a previous review study [8]. From this review and from experience of and feedback from the 2009 influenza pandemic, we identified the requirements for the new system. In response to these requirements, the design of the dashboard features incorporated merits of the national surveillance websites identified in the earlier the review, applied principles of efficient data presentation and dashboard design [16,17], and adopted recommendations from professional information technology consultants. Specific examples of each component were mainly extracted from the influenza surveillance dashboard, which was constructed as a demonstration of these design principles.

In the main webpage and drill-down pages, we implemented the following features for efficient data presentation: (1) provision of information that viewers need quickly and clearly, (2) organization of information to support meaning and usability, (3) minimization of distractions, clichés, and unnecessary embellishments that could create confusion, (4) creation of an aesthetically pleasing viewing experience, and (5) consistency of design for easy data comparison. 

The dashboard displays and synthesizes surveillance data at one glance to provide an overview of disease activity from different data streams. Scrolling of pages was avoided so that users can easily focus on the information from different sources rather than searching for information across different pages or sections. All surveillance raw data were stored in standard data file format to achieve data manipulation simplicity and reduce data processing time during the update and server queries of the dashboard. Several pages allow interactive graphics and information display according to end users’ instruction, and we also allow raw data export in common standard data file formats.

### Data Used for Influenza Surveillance

We used five different types of influenza surveillance data for demonstration of the dashboard, namely, the weekly consultation rate of influenza-like illness reported by general out-patient clinics in Hong Kong, weekly consultation rate of influenza-like illness reported by general practitioners (GPs), weekly influenza virus isolation rate, weekly overall school absenteeism rate, and weekly hospital admission rate of children aged 4 and under with principal diagnosis of influenza. The school absenteeism data was provided by BroadLearning Education (Asia) Ltd, an online electronic school administration system service provider. The other four data streams were extracted from the official website of Centre for Health Protection, Department of Health of the Hong Kong Special Administrative Government [18].

### System Development

The system was designed to allow continual development such as incorporating more surveillance data streams or applying more sophisticated analytical algorithms for aberration detection by modifying specific related components of the internal R programs. Little additional programming was needed for database storage and graphic display with changes in surveillance data sources, for example, addition of data from a new surveillance system.

## Results

The influenza surveillance dashboard is hosted on the server of the School of Public Health, the University of Hong Kong (http://sph.hku.hk/dashboard). Here we present the design patterns [19,20] describing the main features of the surveillance dashboard.

### System Architecture

#### Requirements 

The requirements are that the system be simple, stable, and require few resources. An additional requirement is for efficient data transfer and analysis to facilitate timely surveillance of disease patterns.

#### Design 

A simple 3-tiered structure with database, logical, and presentation layers was adopted. The system was built on open source programming languages. Command line based analyzing software with streamline programming was incorporated to avoid unnecessary computational procedures.

#### Example 

**Figure 1:**
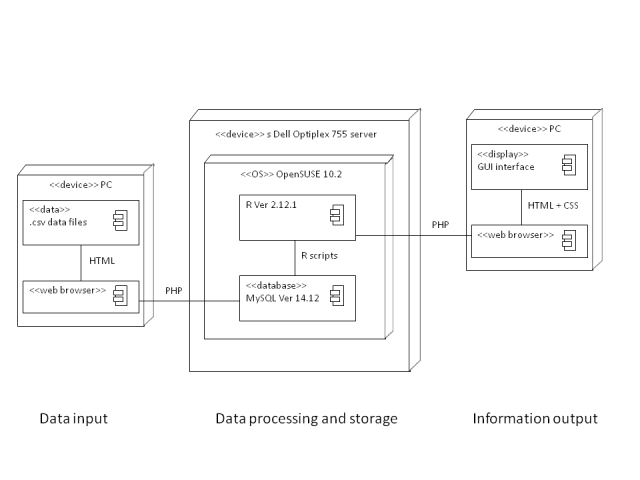
Deployment diagram of the surveillance dashboard design.

For the presentation layer, we used hypertext markup language (HTML) together with cascading style sheets (CSS) and hypertext preprocessor (PHP) Web programming language. For the back-end database, we used the MySQL (structured query language) (Oracle Corporation, Redwood Shores, CA) server. Data in comma separated value (CSV) format were imported to the MySQL server via HTML. The logical layer for data analysis and generating graphical results is based on R version 2.12.1 (R Development Core Team, Vienna, Austria). Figure 1 illustrates the diagrammatic presentation of the system designed in unified modelling language (UML) 2. In this system, each component was connected by specific programming languages via two-way communication. 

### Data Input, Storage, and Extraction

#### Requirements 

A requirement was automated data update to minimize operating resources and human errors. Also required were compatible formats to facilitate data sharing and manipulation in different operating systems and programs.

#### Design 

Format of all raw data were standardized for input and extraction. Surveillance data were stored in a central database connecting to all webpages for easy update. Raw data can be extracted from the database using standard query language (SQL) according to the users’ specification in a user-friendly menu embedded in the webpage.

#### Example 

**Figure 2:**
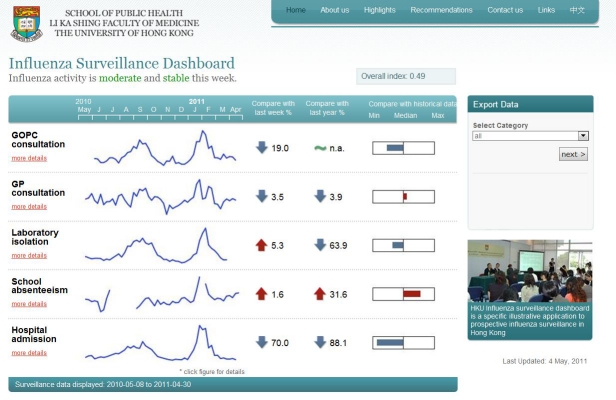
Screenshot of the main page of the influenza surveillance dashboard.

**Figure 3:**
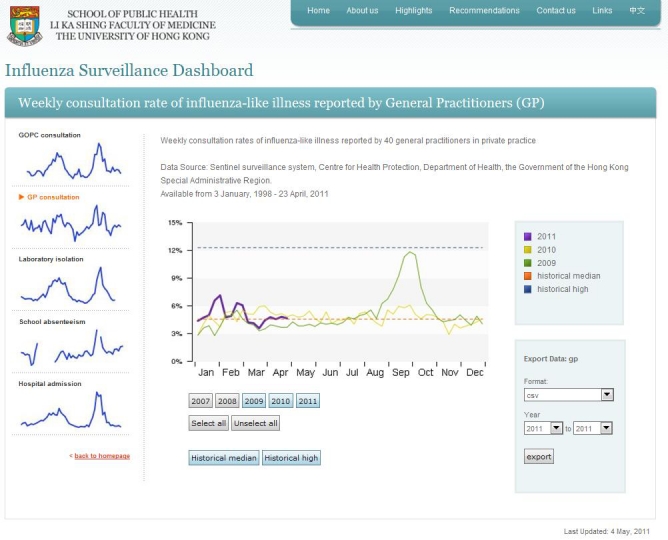
Screenshot of a drill-down page showing the individual data stream (GP consultations) in the influenza surveillance dashboard.

The database can be imported by the one-step data administration system named the “importer,” a tailor-made online data file import system that has been linked to the Internet browser, the back-end server database, and internal PHP and R programs. Once the updated raw data file is ready, the administrative personnel simply upload this file at the importer webpage. The importer will automatically detect the file format and update the central backend MySQL server data in the corresponding data tables. The sparkline and time series image graphs (jpeg format) at the main webpage and drill-down pages will also be updated by the R programs. For data extraction, end users may select specific data stream(s) as well as the time period to extract raw data for their own specific purposes at the main webpage and drill-down pages in comma separated value (CSV) or extensible markup language (XML) format (the panel on the right in Figures 2 and 3).

### Presentation Layer

#### Requirements 

Requirements are display of all surveillance data at one glance in the dashboard to provide a clear overview of disease activity and an interactive display according to users’ specific needs. Also required are graphical alerts to highlight elevated disease activity to achieve effective communications of health information.

#### Design 

Webpages were designed to be content-rich and self-explanatory to present surveillance data streams in clear and simple figures. Distractions were avoided so that surveillance data would stand out on the page. Bright colours or thicker lines were used to indicate high levels or more recent data. Surveillance data streams with increasing trend or at a high level were highlighted by indicators. As surveillance data streams reflect disease activity in different target populations, this allows users to identify elevated disease activity in a certain population. For dynamic graphical display, specific parameters selected by the user in the Web graphical user interface (GUI) will be transferred to the internal programs for generating the most updated figures. Previous and outdated figures will be cleaned up to avoid wastage of server memory.

#### Example

##### Main Page

A screenshot of the influenza surveillance dashboard main page is shown in Figure 2. In this figure, the five influenza surveillance data streams in the last 12 months are shown in panels to allow quick comparison of disease trends. On the left, brief titles for the data streams are given where more details will be shown when they are pointed to. Based on the back-end algorithm, the level (low, moderate, or high) and trend (decreasing, stable, or increasing) of the overall influenza activity are shown on the top left. The overall influenza activity index generated by the dynamic linear model ranging from 0 to 1 is shown in the top right. Different formats of raw data can be extracted using the drop-down menu on the right. 

Other measurements or indicators such as comparisons with past data can be added to provide supplementary information of the individual data streams. Raw data of selected data streams and years can also be exported using the drop-down menu on the right.

##### Individual Surveillance Data Stream Drill-Down Pages

The drill-down page for the corresponding individual data stream will be displayed by clicking the figure for the individual data stream (Figure 3). The drill-down page provides more detailed information, such as data from past years, the historical median, or the high level. Users can toggle specific information to be displayed by clicking the corresponding buttons. A thicker line is used to show data in the current year. A navigator bar on the left allows users to select different surveillance data streams. Further stratification of the data can be incorporated in the drill-down page whenever the data are available. We provided stratification of school absenteeism data by district for illustration.

##### Recommendations Drill-Down Page

**Figure 4:**
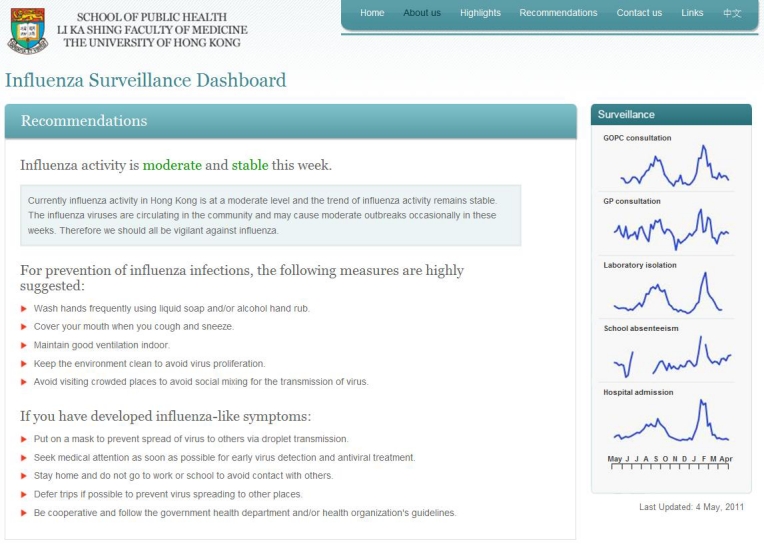
Screenshot of the Recommendations drill-down page in the influenza surveillance dashboard.

As shown in Figure 4, the “Recommendations” drill-down page provides the general public different recommendations and motivates actions for prevention of influenza infection and transmission corresponding to the level and trend of overall influenza activity.

Other drill-down pages provided supplementary information on the sources of the data streams and other related information. The Chinese version of the influenza surveillance dashboard can be assessed by clicking the “Chinese” option at the top right of each page.

## Discussion

We developed an influenza surveillance dashboard that serves as a template to demonstrate efficient synthesization and dissemination of multiple-source surveillance data, which may also be applied to other diseases. Surveillance data from multiple sources can be disseminated efficiently using a dashboard design, which facilitates the translation of surveillance information to public health actions.

Public health surveillance is defined by Thacker as “the ongoing, systematic collection, analysis, and interpretation of health data essential to the planning, implementation, and evaluation of public health practice, closely integrated with the timely dissemination to those who need to know” [21]. Previously, much effort and many resources were required to improve the quality of surveillance data and the accuracy of diagnostic tests. Until the late 1990s, with the rapid development of information technology and the Internet, the timeliness of surveillance data transfer improved. Nevertheless, the methods and targets of data dissemination have been somewhat neglected, probably because communication and graphic design are apparently unrelated to the field of medical science. Indeed, timely and effective health communication is extremely valuable to transfer information to the relevant people who can take appropriate action. In particular, timeliness is always a critical factor for infectious disease containment and control. Comparatively speaking, fewer resources are needed to develop effective data dissemination methods than are required for data collection, quality control, diagnosis, and analysis. Researchers should also keep an eye on data dissemination to maximize the usefulness of the upstream surveillance effort.

A review of national surveillance websites [8] provided evidence that less attention has been paid to designing user-friendly and efficient dissemination of surveillance data. In particular, many websites have presented or highlighted data in report form, which made information more difficult to be identified or synthesized. Long reports with scattered information over pages also made it difficult to grasp the overall situation of disease activity. Also, functions for data retrieval were not commonly available for most websites. Such functions are greatly needed for efficient and timely data sharing. 

The dashboard style can automatically summarize data and present figures of different types in the drill-down pages. Other detailed or supplementary information, such as past records of individual surveillance data streams or recommendations can be available in other drill-down pages. We also incorporated a data export function in standard format (eg, CSV and XML) to facilitate data sharing, which was shown to be important for epidemiological enquiry in the recent human influenza A (H1N1) pandemic.

Moreover, a primary motivation for publicizing surveillance data online should be to allow timely risk communication thereby facilitating disease prevention [22]. As public health awareness has been elevated since the SARS outbreak in 2003, it is expected that sources of surveillance data will only increase. In the influenza surveillance dashboard, we demonstrated how recommendations can be made corresponding to multiple surveillance data so that the collected information can be more efficiently transferred into public health actions.

While more surveillance data streams are being developed, there is a need for developing more sophisticated algorithms for aberration detection. Here, some challenges remain for the development of multivariable analytic algorithms as different surveillance data streams represent situations of different targets at different stages of disease progression. Furthermore, evidence is still lacking about the association between these new surveillance data streams and influenza activity. Whether these data provide more signal or noise is still in doubt due to their nonspecific nature. These data streams will still need to be evaluated and filtered to increase the signal-to-noise ratio before adding them as a routine surveillance practice; thus, they can only serve as supplementary information at the current stage. Other challenges for using combined surveillance data from different sources include the handling of missing data (eg, school absenteeism during school holidays), data provided in different resolutions (daily versus weekly resolutions), and unsynchronized data receiving protocols from different sources, which the algorithm should also be capable of handling. 

Generally speaking, an ideal surveillance website should have comparable hardware and software and a standard user interface, data format, and coding for easy data sharing. The influenza surveillance dashboard demonstrates how different sources of surveillance data streams can be synthesized and efficiently displayed in a dashboard to provide an overview of disease activity as well as each individual source. The influenza surveillance dashboard serves as a template that can provide for efficient dissemination in a similar manner of information about other diseases with multiple surveillance data sources. With a back-end algorithm, indicators or statistics can be generated from individual or all data streams. Other more complicated indicators can also be generated that are tailored to specific disease type by modifying the back-end algorithm.

## Abbreviations

CSS: cascading style sheets
CSV: comma separated value
GP: general practitioner
GUI: graphical user interface
HTML: hypertext markup language
PHP: hypertext preprocessor
SARS: severe acute respiratory syndrome
SQL: standard query language
WHO: World Health Organization
UML: unified modeling language
XML: extensible markup language
